# Eye Tracking Study of Social Intensity on Social Orientation of Autistic Children

**DOI:** 10.3390/bs12090322

**Published:** 2022-09-05

**Authors:** Yajing Zhang, Kun Zhang, Jingying Chen, Lili Liu, Meijuan Luo, Qian Chen, Xiao Zeng, Guangshuai Wang

**Affiliations:** 1 National Engineering Research Center for E-Learning, Faculty of Artificial Intelligence in Education, Central China Normal University, Wuhan 430079, China; 2 National Engineering Research Center of Educational Big Data, Faculty of Artificial Intelligence in Education, Central China Normal University, Wuhan 430079, China; 3 Central China Normal University Wollongong Joint Institute, Central China Normal University, Wuhan 430079, China; 4 School of Computer Science, Wuhan University, Wuhan 430072, China

**Keywords:** eye tracking, social intensity, social scene, joint attention, social orientation, ecological validity

## Abstract

Some previous studies indicate that impaired social attention mainly results in social disorders in autistic children. In the social attention mode of autistic children, social orientation and joint attention are particularly important. The influence of different social intensity and ecological validity on them are worthy of further study. This study used realistic paintings with moderate ecological validity as experimental materials, to design isolated individual scene and social interaction scene, and to explore the impact of social interaction on the social orientation of autistic children. It found that in the scenes without social interaction, the attention patterns of autistic children and typical developing children were the same, while the attention patterns of autistic children were abnormal in the scenes with social interaction. From the eye tracking data, it was shown that the gaze processing process of autistic children was not as smooth as that of typical developing children. Compared with cartoons and other social scenes with low ecological validity, realistic painting could better restore the proportion of real scenes. Moreover, it could reduce the complexity of information which could not be done in real scenes. The findings of this study provide support for training and education of autistic children. Intervention with realistic paintings is conducive to the migration of autistic children.

## 1. Introduction

Autism spectrum disorder (ASD) is a neurodevelopmental disorder. The incidence of ASD has been on the rise since Kanner first described the symptoms in the 1940s [1]. According to the ASD incidence report issued by the Centers for Disease Control and Prevention (CDC) in 2021, there is one child with ASD in every 44 children in the United States, and the prevalence is independent of race [2]. ASD has received more and more attention from the society and has become a hot research field for more and more scholars.

The Diagnostic and Statistical Manual of Mental Disorders (DSM-5), published in 2013, put forward two major symptoms of ASD: social communication and social interaction defects, and restricted and repetitive behavior patterns and interests or activities. Among them, social disorder is one of the core disorders of ASD. Studies have found that these social disorders are mainly caused by impaired social attention [3].

Social attention refers to the processing and treatment of social information, including awareness of people and things in social situations, social orientation, visual search, and joint attention [4]. Social orientation refers to the ability of individuals to autonomously perceive and observe social stimuli occurring in the environment, such as characters, faces, eyes, and body postures [5]. Infants, preschool children, and adults all have social attention orientation. Compared with typical developing (TD) children, autistic children have certain impairments in their social attention and orientation [6].

For non-social stimuli, such as buildings and objects, autistic children and TD children have similar cognitive behaviors [7]. As for social stimuli, such as faces, eyes, and body movements, autistic children and TD children have different visual attention patterns and cognitive performances [8,9,10,11,12].

Yarbus [13] did some experiments creatively with human faces and found that TD individuals had an attentional preference for faces [14], while autistic individuals tended to show “face avoidance” [15]. They have differences in facial perception and facial gaze, as well as differences in their processing strategies for the internal feature areas of the face. In experiments with human face images, TD individuals prefer to look at the eyes in the face, while autistic individuals often show a viewing preference of “more mouths and fewer eyes” [16]. In natural situations, this phenomenon also exists when autistic individuals observe human faces [16]. Some researchers believe that for the gaze process of a face, autistic individuals adopt a “simple mode”, using parts of the brain to process the face [17], while TD individuals use the “social mode” [18].

The research method of a single face deprives the character’s body and other bio-logical information and non-biological information such as objects in the real scene, and cannot simulate the actual living environment well. In order to figure out this problem, the researchers used stimulus materials with social context. The results show that autistic individuals have low gaze preference for people and have a longer gaze for objects [18,19,20] in pictures containing people and objects. Yarbus’ research shows that people will look at the eyes first in the display of human faces. However, the eyes are not selected first when the face and the body are present at the same time. Compared with TD individuals, autistic individuals pay less attention to faces in the scene, and pay more attention to non-social parts (such as body shadows) [20]. In social situations, autistic children spend less time looking at the eye area and more time looking at the body area, and they show low preference for social cues (gaze direction) compared to TD children [21].

Observers follow social cues such as head orientation [22], gestures [23], and other directional instructions [24], and devote the same attention direction or goal [25,26]. This process is called joint attention [27]. The prerequisite for arousing common attention is to produce social orientation. Studies have suggested that the impaired joint attention ability of autistic children is related to the orientation ability to social stimuli [5].

According to studies, the factors that affect the social attention of autistic children include the familiarity with the characters in the stimulus material, the tasks, the age characteristics of the scene, the social intensity of the scene, and the ecological validity.

Compared with strangers, people generally have a faster and more accurate recognition of familiar faces [14]. There is no significant difference in the gaze patterns of familiar faces between autistic children and TD children, while on unfamiliar faces, autistic children have significantly different gaze patterns than TD children [28]. TD children have different processing methods for acquaintances and strangers. On the opposite, autistic individuals have no obvious difference in the gaze patterns of familiar faces and unfamiliar faces [29]. Studies by Yarbus, Birmingham, and others have shown that the distribution pattern of social attention is highly task-dependent. The age characteristics of the characters in the scene will affect the gaze patterns of autistic children. The duration ratio of autistic children’s visits to the face, eyes, and mouth areas of interest in children’s scenes is longer than that of adult scenes, while TD children have the opposite. Compared with adult scenes, autistic children tend to look at faces and backgrounds in children’s scenes [30].

Studies have shown that both autistic individuals and TD individuals pay more attention to scenes with people than scenes without people [31]. In the case of people, the intensity of social information can be measured by activities and social behaviors. Some scholars also use the number of people in the scene to control the intensity of the social information in the scene, the essence of which is to control the complexity of the social behavior in the scene. Among them, the essential difference between the one-person scene and the multi-person scene is that there is no social behavior in the one-person scene, and the intensity of social information is weak. At this time, autistic individuals and TD individuals have similar social attention performance [32,33]. Bimingham [25] controlled the activities of complex real-world scenes and the presence or absence of social behaviors (1 person vs. 3 people). The findings demonstrate that by increasing the intensity of social information, TD adults can increase the gaze of the eyes in the scene. The research of Rigby et al. on autistic adults proved that with the increase of social behavior (the number of people in the scene), the gaze on the face gradually decreased and the gaze on the body and the background area except the person gradually increased [34].

The ecological validity of the scene will also affect the social attention of the observer [35,36]. The closer it is to the real-life scene, the higher its ecological validity is. Current research on visual search is biased toward real scenes, and its purpose is to improve the ecological validity of experiments. But there are a lot of complex information in real scenes, which makes it difficult for autistic children to deal with social stimuli. Instead, researchers simplified the real scenes and used cartoon scenes and stick figure scenes as stimulus materials. Social attention performance of autistic children is better, but its manifestation is more exaggerated, such as increased ratio of eye information in faces, exaggerated facial expressions, simplified body images, which are not conducive to migration to real scenes. Realistic painting is a concrete art in terms of artistic form. It conforms to the observer’s visual experience by reproducing external objects, while processing irrelevant clues such as background to reduce the information complexity of the scene. Its ecological validity lies between real scenes and cartoon and stick figure scenes.

When faced with highly ecologically valid and complex scenes, TD individuals have a gaze preference for characters, especially their faces [25]. In real social scenes, with the increase of social intensity, the fixation of TD children on the mouth is greater than the fixation on the eyes [37], while the conclusion drawn in cartoon and stick figure scenes is on the contrary. Yarbus et al. let adults watch the realistic painting “Unexpected Return” [13]. By increasing the social intensity, they found that the observer’s fixation on the eyes of the characters also increased. The results of some experimental studies have shown that the social attention deficit of autistic children may be related to the ecological validity of the stimulus [38,39]. Delphine et al. [40] found that autistic children used different processing strategies for cartoon faces and real faces. When Van der Geest et al. [39] presented cartoon social scene materials to autistic children, they found that their gaze patterns were similar to those of TD children, paying more attention to cartoon characters and gazing at objective objects. As the complexity of the scene increases, the attention to the characters increases and the gaze to the background does not change significantly. The difference in this result may be that cartoons and other stimulus materials with low ecological validity are not as real as the characters in life, the information complexity is low and less socially threatening to autistic individuals. However, there are few studies on realistic painting scenes between the ecological validity of real scenes and cartoon scenes. For autistic individuals, realistic painting scenes play a transitional role in migrating to real scenes during intervention, and plays an important role in their research.

This paper adopts realistic painting scenes with medium ecological validity, and controls the social intensity by controlling whether the scene has social interaction or not (isolated individuals vs. social interaction), and make some discussions about the influence of social intensity on the social orientation and joint attention of autistic children in realistic paintings.

## 2. Materials and Methods

### 2.1. Participants

A total of 32 autistic children aged 3–6 were recruited from a rehabilitation institution, and 22 TD children aged 3–6 were recruited from a kindergarten in Wuhan. Some participants were unable to complete the experiment due to inattention, turning their heads many times, or being emotional, resulting in the incomplete viewing of the experimental stimulus materials or the severe lack of experimental data. Gaze samples below 70% were considered severely lacking in experimental data, and data from these participants would not be included in the experimental data analysis. Therefore, a total of 13 autistic children and 3 TD children were excluded from the experiment. In the end, 19 autistic children (Experimental group, 14 boys and 5 girls, mean age = 4.51 years) and 19 TD children (Control group, 10 boys and 9 girls, mean age = 4.45 years) were valid participants. They are grouped and matched according to the chronological age.

Before the experiment, all autistic children met: (1) the ASD criteria of the DSM-5; (2) double-blindly diagnosed by two expert physicians in children’s developmental behavior. All TD children were evaluated by the Developmental Scale for Children Aged 0–6 years [41] for evaluating their developmental behavioral level, and all obtained a developmental quotient of more than 80 points (the reference range of developmental quotient: above 130 is excellent, 110–129 is good, 80–109 is moderate, 70–79 is critically low, and less than 70 is mental retardation).

After parental interviews and clinical observations, the two groups of participants were excluded from respiratory diseases, childhood schizophrenia, epilepsy, and other organic brain diseases, and were confirmed to have normal vision (or corrected vision) and normal intelligence. They all had not participated in similar experiments in the previous period, and had never seen this experimental material.

Privacy protection agreements were signed with the institution and the kindergarten. The informed consent was signed with their parents to protect the privacy of these children participating in the experiment. Only relevant data about the participants completing the experimental tasks were collected anonymously. No personally identifiable information or portraits of participants were involved.

### 2.2. Design

A two-factor repeated measurement design was used in this experiment, with the category of participants (ASD vs. TD) as the between-subjects factor, with autistic children as the experimental group and TD children as the control group, to sociability (isolated individuals vs. social interaction) as the within-subject factors. When analyzing eye tracking data, another factor (area of interest) would be added.

Data analysis methods used in the experiments included:

- 2 (subject categories: ASD vs. TD) × 2 (sociality: isolated individual vs. social interaction) mixed analysis of variance (ANOVA) including fixation count and fixation duration was used to explore whether there were differences in the social attention of different participants in different scenarios.
- 2 (subject categories: ASD vs. TD) × 2 (sociality: isolated individual vs. social interaction) × 4 (area of interest: F, B, BG, and ACT) three-factor repeated metric analysis of variance was used to further explore whether there were differences in the attention areas of different participants in different social scenarios.

### 2.3. Stimuli

**Figure 1:**
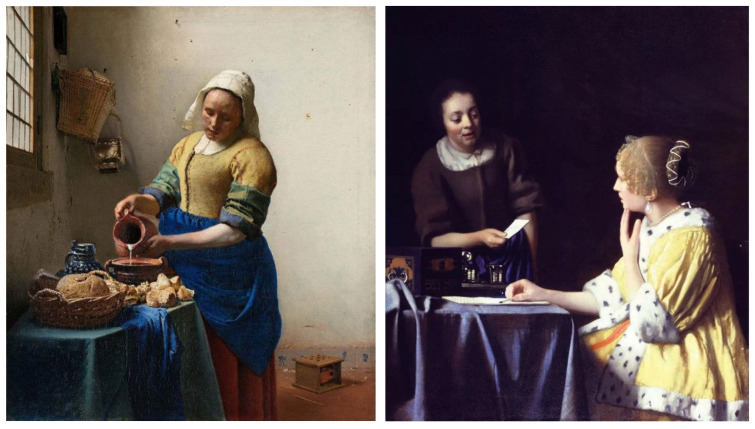
Experimental material picture. (**Left** picture: The Maid Pouring Milk represents an isolated individual scene. **Right** picture: A Lady and Her Maid represents a social interaction scene).

For the selection of experimental stimuli, in order to prevent the interference of children’s training or previous experience, those stimuli that children could understand but had not seen were selected to enhance the effect of the experiment. Paintings that represent common scenes of daily life were good candidates, such as one person pouring water and two people talking. First, 10 realistic paintings depicting scenes of daily life, each with 1 person or 2 people, were selected as alternative materials. Then, 5 specialists in art with a master’s degree or above as art assessors were invited to use the Likert scale to score the paintings for familiarity, interactivity, emotional intensity, and other aspects. According to the scoring result, the pictures of different number of people who were unfamiliar, interactive, emotionally neutral, and with unexaggerated posture were selected, to respectively represent the isolated individual scene and the social interaction scene. Finally, two paintings were selected as the experimental stimuli for this study. As shown in Figure 1, the selected pictures were “The Maid Pouring Milk” and “A Lady and Her Maid” by Vermeer of the Netherlands. Adobe Photoshop CS6 software was used to process the selected pictures so that the width of each picture is 800 pixels, maintaining the aspect ratio of the original picture.

### 2.4. Apparatus

The experimental materials were presented to the participants on an all-in-one computer through Microsoft Visual Studio software, and the touch screen resolution was 1920 × 1080. Tobii Eye Tracker 5 (Tobii, Stockholm, Sweden) was used to record the eye tracking data of the participants when they viewed the experimental material, the sampling frequency of the eye tracker is 90 Hz, gaze angle is 40 × 40 degrees.

### 2.5. Procedure

**Figure 2:**
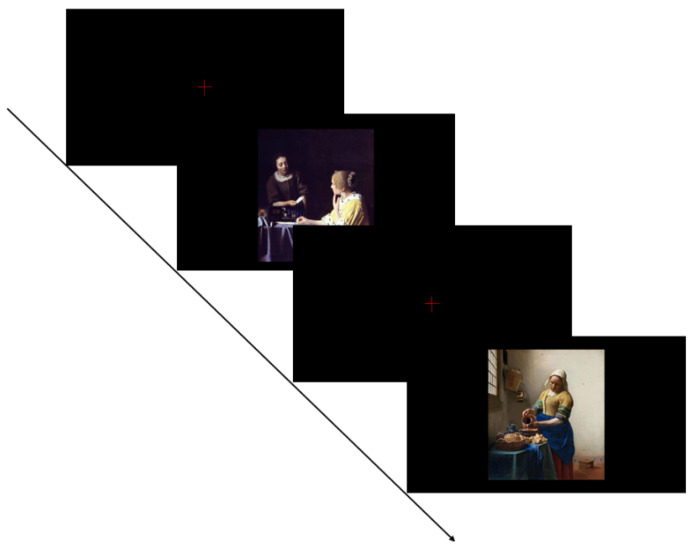
Experimental program example.

The experiment was conducted in a quiet classroom of the special education institution and kindergarten. Each experiment required one participant and two operators. One operator was responsible for controlling the computer program to ensure that it did not interfere with the participant’s viewing. Another operator was responsible for explaining the experiment process. Before the start of the experiment, the operator played with the children sufficiently to gain the children’s trust. TD children were brought into the experimental room by the operator, and did not feel nervous because of the trust established in the early stage. For autistic children, a teacher they were familiar with acted as one of the operators to reduce their anxiety. Parents were not involved in the experiment. In the experiment, each participant was tested individually. Adopt the form of free viewing paradigm. The presentation of experimental procedures is shown in Figure 2.

- The participants sat in front of the computer with their eyes flush with the center of the screen. The distance between their eyes and the screen was about 50–70 cm. Then they were required to gaze at 7 target points on the screen in sequence (a central point, then three peripheral points, then another set of three peripheral points), staring at each point until it disappeared to complete the calibration.
- After calibration, the participants were reminded to watch the experimental materials on the screen. First, a small red “+” would appear on the black screen and hold for 500 ms to attract participants’ attention. Second, stimulus materials were introduced and played in random order, and each one would appear for 5 s. When each piece of material was switched, there would be a 500 ms black screen with a red “+” in the middle to attract participants’ attention. This operation was repeated until the end of the experiment.
- After starting the experiment, there was no instructions during the whole process, experiment adopted the free-to-view paradigm and did not require any tasks from the participants.
- The entire experiment took about 2 min.

### 2.6. Analysis Indicators

Data cleaning mainly dealt with missing values. The missing values in the data were filled with the mean of the data for the same group of participants (ASD or TD).

The collected data from the Tobii Eye Tracker 5 were imported into the OGAMA 5.1 (Opensource software), segmented by the area of interest (AOI), and the fixation count and fixation duration were analyzed. Then statistical analysis was performed using IBM SPSS 27 to discuss their differences.

**Figure 3:**
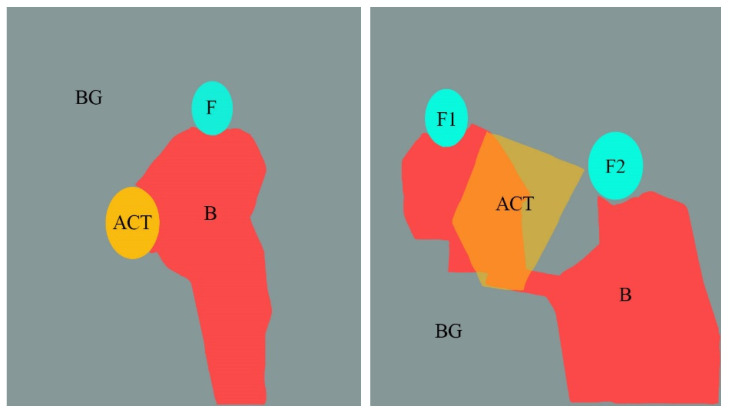
Division of AOI in isolated individual scene (**Left**) and social interaction scene (**Right**). (F: face AOI. B: body AOI. ACT: activity AOI. BG: background AOI).

The AOI is divided in OGAMA software, as shown in Figure 3. Section F is the face AOI (personal face), section B is the body AOI (personal body part), section ACT is the activity AOI (the person’s line of sight is facing the area), section BG is the background AOI (the area excluded the characters and the activity AOI).

The eye tracking indicators used in the experiment included:

- Fixation count (FC): the total number of fixation points of the participant in the target area. In the Tobii Eye Tracker 5, the participant stayed in the target area for more than 100 ms as one fixation.
- Fixation duration (FD): the sum of the participant’s fixation time at all fixation points in the target area. The longer the fixation time, the higher the participant’s attention to the area, and the higher the degree of processing.

## 3. Results

### 3.1. Overall Fixation Count

In order to explore the influence of social interaction in the scene on the processing depth of autistic children and TD children, the fixation counts of two groups were analyzed by 2 (subject categories: ASD vs. TD) × 2 (sociality: isolated individual vs. social interaction) mixed analysis of variance (ANOVA). After Mauchly sphericity test, the result was reported as the corrected result. The degree of freedom in decimal form was the degree of freedom corrected by Green-house-Geisser. The following data processing methods were the same.

The overall fixation counts of two groups of children in different scenes were shown in Table 1.

**Figure 4:**
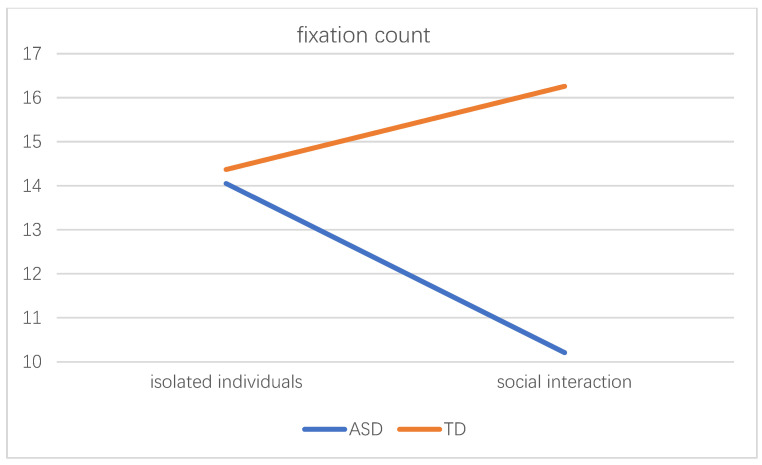
The interaction diagram of the subject category and the scene.

The experimental results showed that the main effect of the subject category was significant, F(1,36) = 10.500, *p* = 0.003 < 0.01. In terms of fixation count in the entire picture, the social interaction between the subject category and the scene was significant, F(1,36) = 8.520, *p* = 0.006 < 0.01. The interaction diagram is shown in Figure 4.

Further analysis of its simple effect, there was no significant difference in scene sociality among autistic children (F(1,36) = 3.92, *p* = 0.055 > 0.05) and TD children (F(1,36) = 2.96, *p* = 0.094 > 0.05). In the scene of isolated individual scene, there was no difference in the fixation count caused by the differences in the subject category, F(1,36) = 0.05, *p* = 0.822 > 0.05. When the scene had social interaction, the fixation count in the picture caused by the difference in the subject category was different, F(1,36) = 18.97, *p* = 0.000 < 0.01. TD children had more fixation count than autistic children.

### 3.2. Fixation Duration

In order to explore whether social interaction has an impact on children’s fixation duration, and whether there are two types of children’s attention and processing levels in social interaction scenes, the overall fixation duration is supposed to be analyzed. In order to further explore children’s attention distribution patterns, the fixation duration of different areas of interest needs to be analyzed.

#### 3.2.1. Overall Fixation Duration

A 2 (subject categories: ASD vs. TD) × 2 (sociality: isolated individual vs. social interaction) repeated metric analysis of variance was performed on the fixation duration of the entire picture. The overall fixation duration of two groups of children in different scenes was shown in Table 2. The results showed that there was no interaction between the subject category and the sociality of the scene, F(1,36) = 0.833, *p* = 0.367 > 0.05. The main effect of the subject category was extremely significant and unrelated to whether the scene had social interaction or not, F(1,36) = 9.714, *p* = 0.004 < 0.01. The fixation duration of TD children was longer than that of autistic children. There were differences in the social nature of the factors within the subjects, F(1,36) = 5.751, *p* = 0.022 < 0.05, and the fixation duration of the isolated individual scene was longer than that of the social interaction scene.

#### 3.2.2. Fixation Duration of AOI

**Figure 5:**
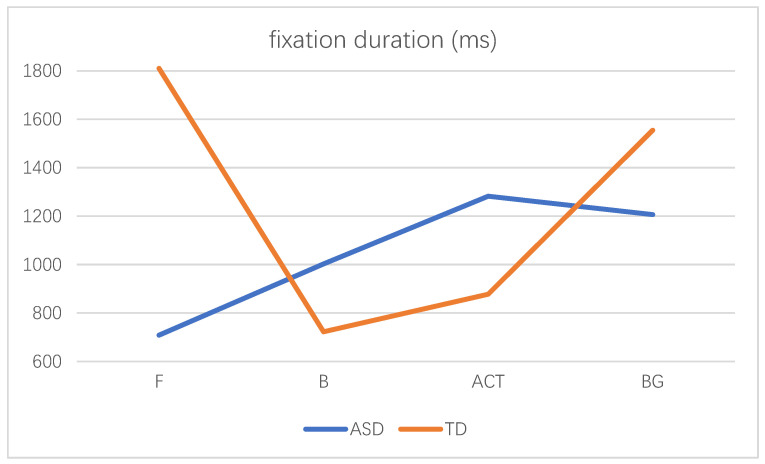
The interaction diagram of the subject category and AOI in the social interaction scene.

In order to explore the difference in attention distribution and processing mode between autistic children and TD children in realistic painting scenes, a 2 (subject categories: ASD vs. TD) × 2(sociality: isolated individual vs. social interaction) × 4 (AOI: F, B, BG and ACT) three-factor repeated metric analysis of variance was performed. The fixation duration of AOI of autistic children and TD children in different scenes was shown in Table 3. The results showed that the main effect of the AOI was significant, F(3,34) = 8.728, *p* = 0.000 < 0.01. The social interaction between AOI and the scene was significant, F(3,34) = 9.437, *p* = 0.000 < 0.01. The complexity of AOI and subject category was significant, F(3,34) = 3.378, *p* = 0.029 < 0.05. The interaction between the subject category, the sociality of the scene, and AOI were significant, F(3,34) = 3.993, *p* = 0.015 < 0.05. A simple interaction effect analysis was further carried out. In the isolated individual scene, the interaction between the subject category and AOI was not significant, F(3,108) = 0.53, *p* = 0.665 > 0.05. In the social interaction scene, the interaction between the subject category and AOI was significant, F(3,108) = 4.23, *p* = 0.007 < 0.01. The interaction diagram was shown in Figure 5. Further analysis of its simple effect, in the social interaction scene, only in the face AOI, the subject category had a significant difference, F(1,36) = 20.02, *p* = 0.000 < 0.01, the fixation duration of TD children was longer than the autistic children. There were no significant differences in the subject category in the fixation duration of other AOI (B: F(1,36) = 0.88, *p* = 0.354 > 0.05; ACT: F(1,36) = 1.16, *p* = 0.289 > 0.05; BG: F(1,36) = 1.09, *p* = 0.303 > 0.05).

**Figure 6:**
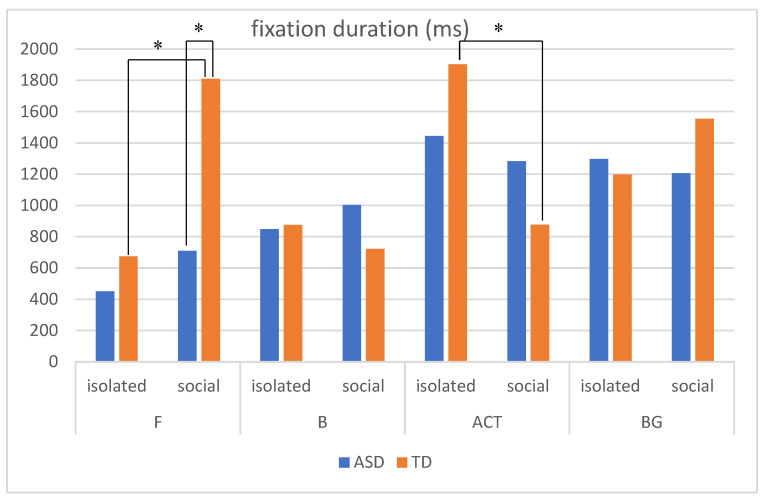
Fixation duration of autistic children and TD children in different scenes (* indicates a significant difference in means using pairwise comparisons, *p* < 0.05. There is significant difference between two groups in the face AOI in social interaction scene. There is no significant difference between the fixation duration of each AOI in different social scenes of autistic children. TD children has significant differences in the fixation duration of different social scenes in both the face AOI and activity AOI).

As shown in Figure 6, for autistic children, there was no significant difference in the fixation duration generated by different social scenes in each AOI. For TD children, there was no significant difference in the fixation duration between different social scenes in the body AOI and background AOI. However, in the face AOI, the fixation duration of the isolated individual scene was significantly less than that of social interaction scene (t = −4.74, *p* = 0.000 < 0.01). In the activity AOI, the fixation duration of the isolated individual scene was significantly longer than that of social interaction scene (t = 3.782, *p* = 0.001 < 0.01). With the increase in the number of scenes, attention of TD children to the activity AOI decreased and their attention to the face AOI increased, while the social changes of the scene did not have a significant impact on the attention distribution of autistic children.

## 4. Discussion

In order to intuitively see the attention distribution of autistic children and TD children when watching different social scenes, OGAMA software was used to generate eye tracking heat maps and eye movement trajectories. In the heat maps, there were three colors: red, orange, and green. The darker the color (the redder), the longer the fixation duration of the subject in the area, and the more fixation count. The lighter the color, the fewer fixation count and the shorter the fixation duration, as shown in Table 4. The eye trajectory chart recorded the order of eye movement, and then analyzed the gaze patterns of autistic children and TD children. The first fixation point appeared randomly, which was related to the transformation of the picture, as shown in Table 5.

In the isolated individual scene, there was no significant difference in the overall fixation count of two groups of children. Though the overall fixation duration of TD children was longer than that of autistic children, and there was no significant difference in the attention distribution in each AOI. The fixation preferences of TD children and autistic children were the same, and there were two eye-tracking hot spots: the face AOI and the gaze direction AOI. In the eye trajectory diagram, both TD children and autistic children first noticed social stimuli (faces), and then followed social cues to produce joint attention. But the difference was that after the TD children had the joint attention, they focused their attention on the objects on which the characters produce actions. In addition to the objects in their hands, autistic children also searched for objects on the table and on the wall. This showed that both autistic children and TD children had social orientation and joint attention, but autistic children had difficulty detecting the intentions of the characters in the scene in time, and needed more revisiting and searching, while the gaze process of TD children was relatively smooth. When there was no social interaction in the scene, the performance of autistic children’s social orientation and common attention in the realistic painting scene was consistent with the research results in the real scene [42]. Autistic children noticed the same stimuli as TD children but processed the information in different ways [43].

In social interaction scene, the overall fixation count, overall fixation duration, and fixation duration on face AOI of TD children were greater than those of autistic children. Through a *T*-test, the hypothesis was verified that the possibility of autistic children looking at faces was less than TD children when the scene had social interaction [18]. When there was social interaction in the scene, the processing depth, attention, and social orientation of autistic children were different from those of TD children. Consistent with the previous experimental results [44], there was no significant difference in the fixation duration of the two groups of children between the character’s body and the background AOI. It was indicated that there was little difference between the two groups of children’s processing modes of the characters’ bodies and backgrounds in social scenes and was unrelated to ecological validity of the scenes. The eye tracking hotspot of TD children was the face AOI, while that of autistic children was concentrated in the middle area where the two people’s eyes met. Compared to TD children, autistic children tended to focus on different aspects in the same situation [45,46]. The scenes with higher ecological validity might inhibit their attention to the characters. In the eye movement trajectory, autistic children had no rules to follow and had fewer fixation count. Most of their fixation trajectory focused on the body and the irrelevant information about the background. The gaze trajectory of TD children formed a triangle around the two characters and the communication area between them. Compared with autistic children, they had more fixation count in the face AOI. When there was social interaction in the scene, the ability of autistic children to process social orientation and common attention was greatly reduced. The two characters in the scene pictures which was given in this study were in a state of communicating with each other and the transmission of social information mainly relied on social stimuli such as faces. TD children focused more on the faces of the characters after searching the middle area of the two characters for a short time, while autistic children searched and looked at the middle area of the two characters. The processing ability of autistic children at this time was in an abnormal state from the heat map or the trajectory map.

Studies had shown that in real social scenes, the overall processing of faces by autistic children were different from those of TD children [20]. Consistent with the results of this study using realistic painting materials, it was found that there was no significant difference in the gaze between autistic children and TD children in cartoon scenes with or without social interaction [47]. The difference between the cartoon scene and the real scene might be related to the exaggerated expression of the cartoon. Since the performance of the characters in the realistic paintings was based on the real situation as much as possible, the conclusion of highly consistent with the real scene could be drawn. That was to say, TD children had greater ability to process human faces than autistic children in realistic paintings with medium ecological validity.

The overall fixation duration of the isolated individual scene was longer than that of the social interaction scene. As the sociality of the scene increased, the information complexity of the scene also increased. It might lead to a decrease in interest in attention and a corresponding decrease in the degree of processing. However, for autistic children, there was no significant difference between the two types of scenes in the fixation duration of each AOI. For TD children, after increasing social interaction in the scene, the fixation duration of the face AOI increased, but the fixation duration of the activity AOI decreased. This was consistent with Yarbus’s research conclusions on normal adults watching “Un-expected Return” [13], indicating that the attention distribution pattern of 3–6 years old TD children in social scenes was basically the same as that of adults.

In general, TD children had more regular eye movements and a smoother gaze. The key point of their eye movements was the social stimuli in the scene. The eye movement trajectory of autistic children was disordered, often jumped out of social stimuli and paid attention to non-social stimuli. Moreover, they were different from TD children in understanding intentions. Some researchers pointed out that children’s visual preferences and the reduction of their gaze on the entire scene and face would have a significant impact on the social cues obtained during the development process [45]. It might promote the separation of their social cognitive skills and face perception abilities. In the intervention of autistic children, attention should be paid to the intervention of social orientation and intention understanding. By training autistic children to focus more on faces during social interactions, it would be possible to improve their social attention ability.

## 5. Conclusions

The main conclusions of this study are as follows:

- In the isolated individual scene, the visual processing and attention distribution of autistic children and TD children are consistent.
- In scenes with social interaction, the processing depth, attention to the scene, and social orientation ability of autistic children are different from those of TD children.
- When adding social interaction to the scene, TD children pay more attention to the faces of the characters and pay less attention to the activity area, while autistic children have no such difference.
- TD children have a smoother gaze trajectory toward the scene. Their ability to deal with social stimuli and understanding of characters’ intentions is stronger than that of autistic children.

## Figures and Tables

**Table 1:** Overall fixation counts of experimental group (autistic children) and control group (TD children) in different scenes.

	Experimental Group		Control Group	
	M	SD	M	SD
Isolated individual scene	14.05	5.59	14.37	3.17
Social interaction scene	10.21	6.35	16.26	3.60

**Table 2:** Overall fixation duration of experimental group (autistic children) and control group (TD children) in different scenes (unit: ms).

	Experimental Group		Control Group	
	M	SD	M	SD
isolated individual scene	4039.47	1139.78	4651.47	654.01
social interaction scene	3136.37	1600.97	4255.42	1195.23

**Table 3:** Fixation duration of experimental group (autistic children) and control group (TD children) in different scenes. (Unit: ms).

		Experimental Group		Control Group	
		M	SD	M	SD
F Face AOI	isolated	450.00	568.16	674.16	491.33
	social	709.11	988.55	1810.37	920.29
B Body AOI	isolated	848.26	910.49	875.63	718.94
	social	1002.89	1062.86	722.68	516.91
ACT Activity AOI	isolated	1443.74	1318.04	1903.11	988.28
	social	1282.63	1644.62	877.53	648.31
BG Background AOI	isolated	1297.47	1479.42	1198.58	1067.85
	social	1206.16	921.86	1554.53	878.65

**Table 4:** Heat maps of experimental group (autistic children) and control group (TD children) looking at different scenes.

	Experimental Group	Control Group
isolated individual scene	** 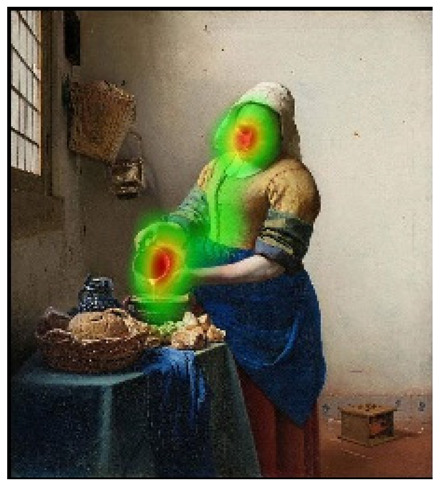 **	** 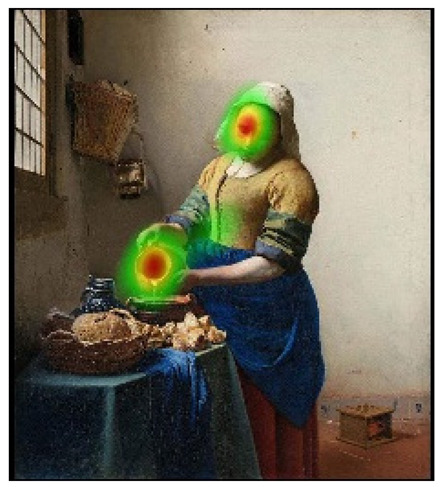 **
social interaction scene	** 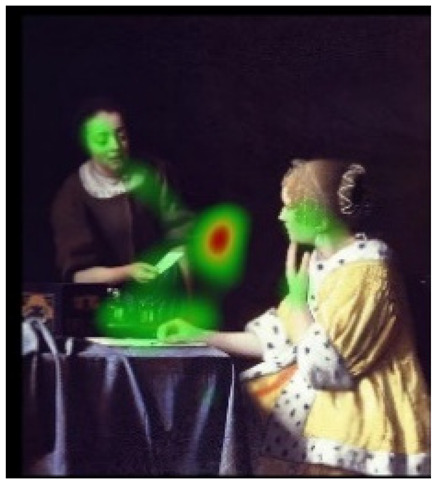 **	** 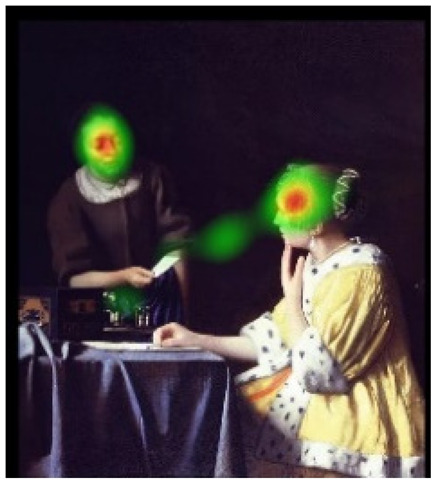 **

**Table 5:** Eye tracking diagrams of experimental group (autistic children) and control group (TD children) looking at different scenes.

	Experimental Group	Control Group
isolated individual scene	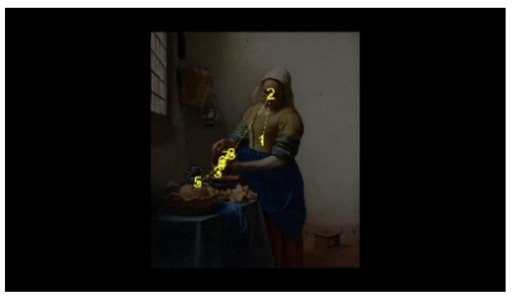	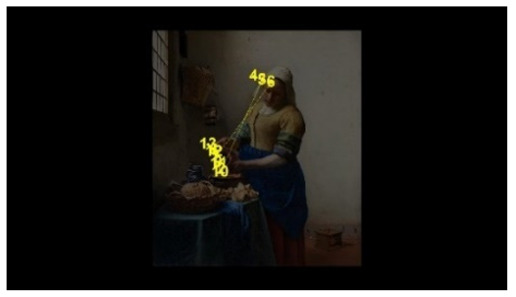
	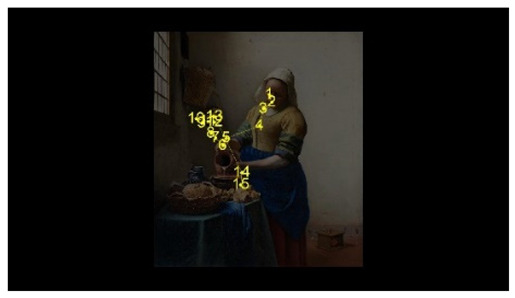	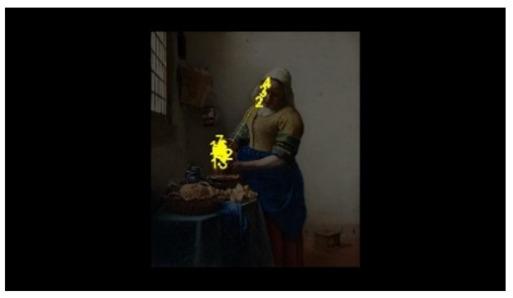
isolated individual scene	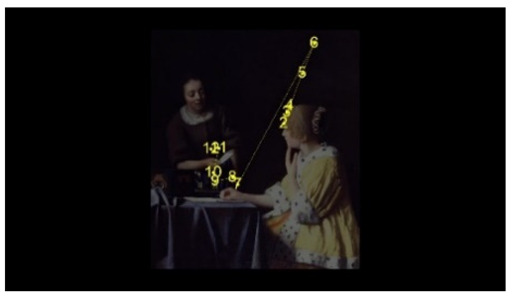	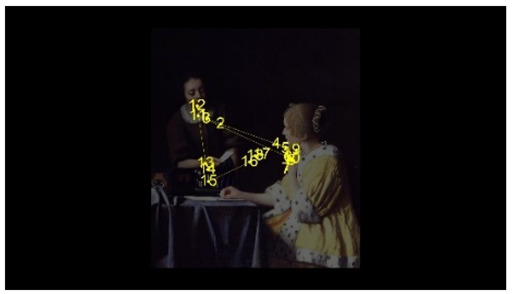
	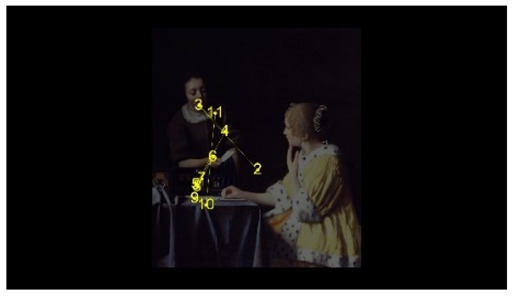	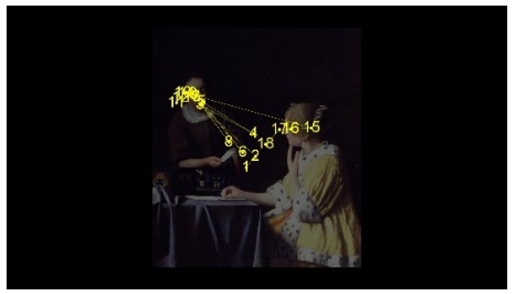

## Data Availability

The experimental data generated during the study are not publicly available due to privacy and ethical restrictions.

## References

[1] Kanner L. (1943). Autistic disturbances of affective contact. Nervous Child.

[2] Maenner M.J., Shaw K.A., Bakian A.V., Bilder D.A., Durkin M.S., Esler A., Furnier S.M., Hallas L., Hall-Lande J., Hudson A. (2021). Prevalence and Characteristics of Autism Spectrum Disorder Among Children Aged 8 Years—Autism and Developmental Disabilities Monitoring Network, 11 Sites, United States, 2018. Morbidity and mortality weekly report. Surveill. Summ..

[3] Freeth M., Bugembe P. (2019). Social partner gaze direction and conversational phase; factors affecting social attention during face-to-face conversations in autistic adults?. Autism.

[4] Hessels R., Holleman G., Cornelissen T., Hooge I., Kemner C. (2018). Eye contact takes two—autistic and social anxiety traits predict gaze behavior in dyadic interaction. J. Exp. Psychopathol..

[5] Dawson G., Meltzoff A.N., Osterling J., Rinaldi J., Brown E. (1998). Children with autism fail to orient to naturally occurring social stimuli. J. Autism Dev. Disord..

[6] Reisinger D.L., Shafer R.C., Horn P.S., Hong M.P., Pedapati E.V., Dominick K.C., Erickson C.A. (2020). Atypical social attention and emotional face processing in autism spectrum disorder: Insights from face scanning and pupillometry. Front. Integr. Neurosci..

[7] Trepagnier C., Sebrechts M.M., Peterson R. (2002). Atypical face gaze in autism. Cyber Psychol. Behav..

[8] Franchini M., Glaser B., Wood D.W.H., Gentaz E., Eliez S., Schaer M. (2017). Social orienting and joint attention in preschoolers with autism spectrum disorders. PLoS ONE.

[9] Ristic J., Mottron L., Friesen C.K., Iarocci G., Burack J.A., Kingstone A. (2005). Eyes are special but not for everyone: The case of autism. Cogn. Brain Res..

[10] Pelphrey K.A., Morris J.P., Michelich C.R., Allison T., Mc Carthy G. (2005). Functional anatomy of biological motion perception in posterior temporal cortex: An FMRI study of eye, mouth and hand movements. Cereb. Cortex.

[11] Tardif C., Laine F., Rodriguez M., Gepner B. (2007). Slowing down presentation of facial movements and vocal sounds enhances facial expression recognition and induces facial–vocal imitation in children with autism. J. Autism Dev. Disord..

[12] Saitovitch A., Bargiacchi A., Chabane N. (2013). Studying gaze abnormalities in autism: Which type of stimulus to use?. Open J. Psychiatry.

[13] Yarbus A.L. (1965). Eye Movements and Vision (B. Haigh, Trans.).

[14] Jan T., Stefan V.S. (2006). Faces capture attention: Evidence from inhibition of return. Vis. Cogn..

[15] Chita-Tegmark M. (2016). Social attention in ASD: A review and meta-analysis of eye-tracking studies. Res. Dev. Disabil..

[16] Chita-Tegmark M. (2016). Attention allocation in ASD: A review and meta-analysis of eye-tracking studies. Rev. J. Autism Dev. Disord..

[17] Hall G.B.C., Szechtman H., Nahmlas C. (2003). Enhanced salience and emotion recognition in autism: A pet study. Am. J. Psychiatry.

[18] Guillon Q., Hadjikhani N., Baduel S., Roge B. (2014). Visual social attention in autism spectrum disorder: Insights from eye tracking studies. Neurosci. Biobehav. Rev..

[19] Papagiannopoulou E.A., Chitty K.M., Hermens D.F., Hickie I.B., Lagopoulos J. (2014). A systematic review and meta-analysis of eye-tracking studies in children with autism spectrum disorders. Soc. Neurosci..

[20] Falckytter T., Hofsten C.V. (2011). How special is social looking in ASD: A review. Prog. Brain Res..

[21] Aldaqre I., Paulus M., Sodian B. (2014). Referential gaze and word learning in adults with autism. Autism.

[22] Parish-Morris J., Pallathra A.A., Ferguson E., Maddox B.B., Pomykacz A., Perez L.S., Bateman L., Pandey J., Schultz R.T., Brodkin E.S. (2019). Adaptation to different communicative contexts: An eye tracking study of autistic adults. J. Neurodev. Disord..

[23] Del Bianco T., Mazzoni N., Bentenuto A., Venuti P. (2018). An investigation of attention to faces and eyes: Looking time is task-dependent in autism spectrum disorder. Front. Psychol..

[24] Pelphrey K.A., Sasson N.J., Reznick J.S., Paul G., Goldman B.D., Piven J. (2002). Visual scanning of faces in autism. J. Autism Dev. Disord..

[25] Birmingham E., Bischof W.F., Kingstone A. (2008). Social attention and real—world scenes: The roles of action, competition and social content. Q. J. Exp. Psychol..

[26] Bayliss A.P., Tipper S.P. (2006). Predictive gaze cues and personality judgments: Should eye trust you?. Psychol. Sci..

[27] Mundy P., Neal A.R. (2000). Neural plasticity, joint attention, and a transactional social—orienting model of autism. Int. Rev. Res. Ment. Retard..

[28] Nuske H.J., Vivanti G., Dissanayake C. (2014). Reactivity to fearful expressions of familiar and unfamiliar people in children with autism: An eye-tracking pupillometry study. J. Neurodev. Disord..

[29] Sterling L., Dawson G., Webb S., Murias M., Munson J., Panagiotides H., Aylward E. (2008). The role of face familiarity in eye tracking of faces by individuals with autism spectrum disorders. J. Autism Dev. Disord..

[30] Wei L., Yu Y., Liu R., Cheng Y. (2020). Age characteristics of scenes affect gaze patterns in children with autism spectrum disorder. Stud. Psychol. Behav..

[31] Frazier T.W., Strauss M., Klingemier E.W., Zetzer E.E., Hardan A.Y., Eng C., Youngstrom E.A. (2017). A meta-analysis of gaze differences to social and nonsocial information between individuals with and without autism. J. Am. Acad. Child Adolesc. Psychiatry.

[32] Elsabbagh M., Bedford R., Senju A., Charman T., Pickles A., Johnson M.H. (2014). What you see is what you get: Contextual modulation of face scanning in typical and atypical development. Soc. Cogn. Affect. Neurosci..

[33] Freeth M., Chapman P., Ropar D., Mitchell P. (2010). Do gaze cues in complex scenes capture and direct the attention of high functioning adolescents with ASD? Evidence from eye tracking. J. Autism Dev. Disord..

[34] Rigby S.N., Stoesz B.M., Jakobsoa L.S. (2016). Gaze patterns during scene processing in typical adults and adults with autism spectrum disorders. Res. Autism Spectr. Disord..

[35] Smilek D., Birmingham E., Cameron D., Bischof W., Kingstone A. (2006). Cognitive ethology and exploring attention in real—world scenes. Brain Res..

[36] Hill J.L., Patel S., Xue G., Seyedali N.S., Bachevalier J., Sereno A.B. (2010). Social orienting: Reflexive versus voluntary control. Vis. Res..

[37] Speer L.L., Cook A.E., Mc Mahon W.M., Clark E. (2007). Face processing in children with autism: Effects of stimulus contents and type. Autism.

[38] Hanley M., Mc Phillips M., Mulhern G., Riby D.M. (2013). Spontaneous attention to faces in Asperger syndrome using ecologically valid static stimuli. Autism.

[39] van der Geest J.N., Kemner C., Verbaten M.N., van Engeland H. (2002). Gaze behavior of children with pervasive developmental disorder toward human faces: A fixation time study. J. Child Psychol. Psychiatry.

[40] Delphine B.R., Ce’cilie R., David D.F., Andreia S., Brigitte A., Christine D. (2008). Typical emotion processing for cartoon but not for real faces in children with autistic spectrum disorders. J. Autism Dev. Disord..

[41] National Health Commission of the People’s Republic of China Developmental Scale for Children Aged 0–6 Years. WS/T 580-2017. http://www.nhc.gov.cn/wjw/pqt/201710/8e070f8482144cae97088668f0dfe25a.shtml.

[42] Schultz R.T., Gsuthier I., Klin A., Fullbright R.K., Anderson A.W., Volkmar F., Skudlarski P., Lacadie C., Cohen D.J., Gore J.C. (2000). Abnormal ventral temporal cortical activity during face discrimination among individuals with autism and asperger ayndrome. Arch. Gen. Psychiatry.

[43] Dapretto M., Davies M.S., Pfeifer J.H., Scott A.A., Sigman M., Bookheimer S.Y., Iacoboni M. (2005). Understanding emotions in others: Mirror neuron dysfunction in children with autism spectrum disorders. Nat. Neurosci..

[44] Riby D., Hancock P. (2008). Viewing it differently: Social scene perception in willimis syndrome and autism. Neuropsychologia.

[45] Freeth M., Ropar D., Chapman P., Mitchell P. (2010). The eye gaze direction of an observed person can bias perception, memory, and attention in adolescents with and without autism spectrum disorder. J. Exp. Child Psychol..

[46] Robain F., Kojovic N., Solazzo S., Glaser B., Franchini M., Schaer M. (2021). The impact of social complexity on the visual exploration of others’ actions in preschoolers with autism spectrum disorder. BMC Psychol..

[47] Rice K., Moriuchi J.M., Jones W., Klin A. (2012). Parsing heterogeneity in autism spectrum disorders: Visual scanning of dynamic social scenes in school-aged children. J. Am. Acad. Child Adolesc. Psychiatry.

